# Microbiome signatures correlate with diet-mediated ADHD symptom reduction

**DOI:** 10.1080/19490976.2026.2659400

**Published:** 2026-04-16

**Authors:** Saartje Hontelez, Martin Guthrie, Tim Stobernack, Peter van Baarlen, Céline Rousseau, Marco P. Boks, Rob Rodrigues Pereira, Jos Boekhorst, Michiel Kleerebezem

**Affiliations:** a Department of Animal Sciences, Host-Microbe Interactomics, Wageningen University and Research, Wageningen, The Netherlands; b Department of Psychiatry, Brain Center University Medical Center Utrecht, University Utrecht, Utrecht, The Netherlands; c Medical Centre Kinderplein, Rotterdam, The Netherlands

**Keywords:** Gut microbiome, attention deficit hyperactivity disorder (ADHD), few foods diet (FFD), multi-omics

## Abstract

Attention-deficit hyperactivity disorder (ADHD) is one of the most common childhood neuropsychiatric conditions. Both (epi)genetic and environmental factors are suggested to contribute to the etiology of ADHD. In the last decade, nutrition has received considerable attention as a potential environmental factor triggering ADHD behavior, particularly applying a few-foods diet (FFD) has been shown to elicit considerable behavioral improvements. These studies are observational rather than investigating underlying molecular mechanisms. The present study included 79 children (boys aged 8–10) with ADHD following a progressive, i.e., increasingly restrictive, FFD diet for 5 weeks. Minimally invasive samples (feces, urine, blood, and buccal swabs) were collected before and after the intervention to obtain a multi-omics perspective of the dietary responses in the participating children. For 63% of the participating children, a more than 40% behavior score improvement was observed, with an average improvement of 73%. The strength of diet-induced changes in ADHD symptoms among children was significantly associated with the gut microbiome composition, particularly when analyzing species-stratified abundance profiles of previously characterized gut–brain modules in the fecal metagenomic data. While integrative multi-omics analysis did not identify composite signatures linked to symptom changes, the strongest multi-omics signal confirmed compliance with the dietary intervention. Our findings implicate a role of the gut microbiome and its metabolic capacity to communicate with the central nervous system in children with food-associated ADHD.

## Introduction

Attention-deficit hyperactivity disorder (ADHD) is one of the most common childhood neuropsychiatric conditions, with an estimated 6% prevalence worldwide.^1^ In Europe, including the Netherlands, the prevalence estimates range from 3% to 5%.^2^ Notably, the prevalence of ADHD in children and adolescents was reported to have increased from 6.1 to 10.2 percent between 1997 and 2016 in the USA.^3^ ADHD is typically characterized by a combination of inattentive, hyperactive and impulsive behavior^4^ and is an impairing condition that is burdensome for child, family and society. Notably, ADHD behavior symptoms are highly variable, with considerable variations in the relative degree of inattentive and hyperactive behaviors.^2^

Although the cause(s) underlying ADHD is (are) still unclear, it is commonly accepted that both (epi)genetic and environmental factors contribute to the etiology of ADHD.^5^ An environmental factor that has received considerable attention in the last decade is nutrition. Clinical trials studying the effects of food ingredients and/or additives on ADHD symptom severity have reported small and often inconsistent or clinically irrelevant effects on behavior. Conversely, studies applying a diet that allows only a few foods, the so-called few-foods diet (FFD), resulted in a clinically relevant reduction of ADHD symptoms in children following the FFD,^6^ resulting in behavioral improvements of at least 40% in 50%–64% of children with ADHD.^7-9^ Considering the evidence for the effect of an FFD on ADHD symptoms, conclusions from observational rather than mechanistic studies, we initiated the ‘Biomarker Research in ADHD: the Impact of Nutrition’ (BRAIN) study with the aim of investigating the mechanisms underlying the behavioral improvements after following an FFD. In this open-label trial, 63% of the participants responded with behavioral improvements of at least 40% after following the FFD, with an average improvement of 73%. The decrease in parent-reported ADHD symptoms was correlated with an activity increase in the precuneus region of the brain during performance of a response-inhibition task.^10^

Since brain function can be affected by the microbiome,^11^ which in turn is strongly affected by diet,^12-14^ it is conceivable that the microbiota‒gut‒brain (MGB) axis is involved in the mechanism of action underlying the behavioral improvements observed in children with ADHD after following the FFD. This finding suggests a role for (microbial) metabolites as molecular intermediates and a potential role for host genetics, which could be addressed though a multi-omics approach. Moreover, the well-established personalized composition of the gut microbiome^15^ may partially explain the large variation observed in behavioral responses in children with ADHD when subjected to an FFD intervention.

The primary aim of this study is to test the hypothesis that ARS score changes during FFD are linked to changes in phenylalanine and tyrosine (and derivatives) levels in blood and urine^16^ and a potential role for 21 gut microbial genes involved in phenylalanine and tyrosine metabolism. As a secondary aim, this study employs an explorative multi-omics approach, to identify associations between diet-induced ARS changes and MGB parameters (i.e., the gut microbiota composition and functionality, blood and urine metabolome, blood immune cell transcriptome and [epi]genetic background). While the type of intervention, i.e., a diet strictly limited to a small group of food ingredients, does not allow for a straightforward double-blind placebo-controlled study, the longitudinal setup and direct molecular readouts allow quantification of the role of the gut microbiota composition and genetic content and allow us to assess whether specific host parameters act as mediators between diet and ADHD symptoms.

## Results

**Figure 1.**
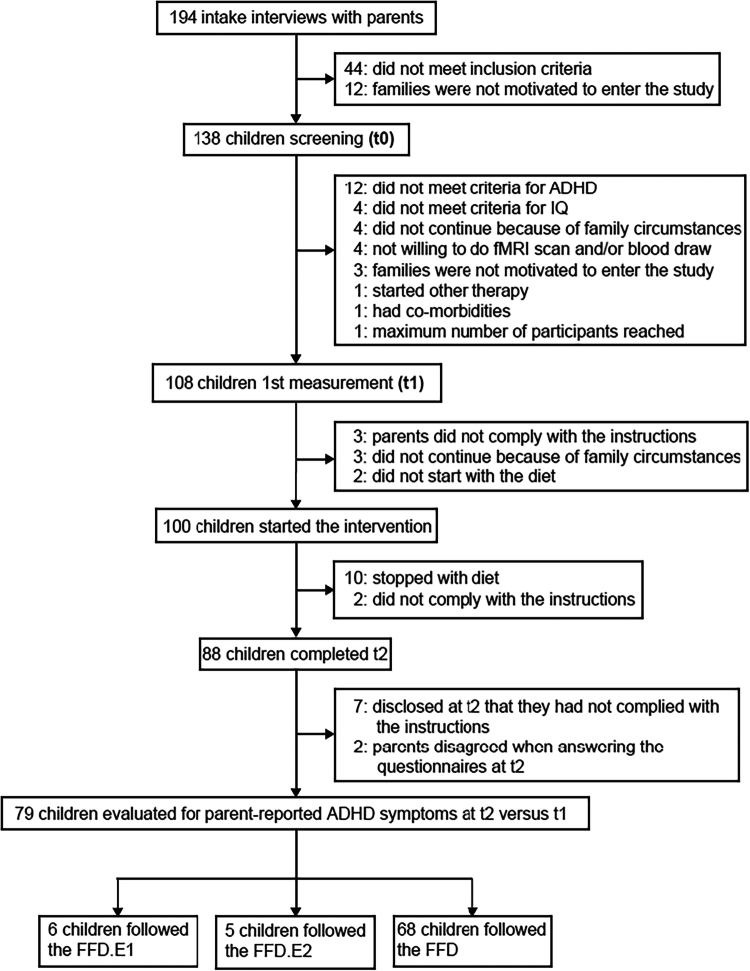
Flowchart shows BRAIN study participant progress: 194 intake interviews, 79 children evaluated. Study flow from start to finish.

**Figure 2.**
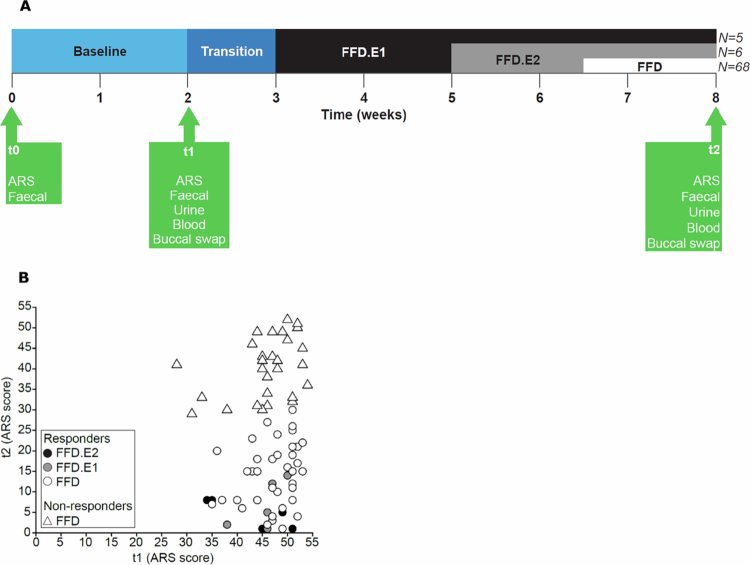
Two panel figure: study design timeline and scatter plot of ARS scores before and after diet intervention.

Of the 100 BRAIN study participants, 21 were excluded owing to non-compliance, or stopped prematurely with the diet (Figure 1). The inclusion/exclusion criteria are reported in the methods section. The habitual diet before the start of the study was not recorded. During the progressive reduction of allowed food ingredients (progressing from FFD-E1 to FFD-E2 to FFD; see methods for details) over a period of 5 weeks, six children responded favorably to the FFD.E1 and five children responded to the FFD.E2 (Figure 2). For those children who showed a favorable response, the diet was not further restricted. For 68 children, the FFD.E1 and FFD.E2 were not effective, therefore these children proceeded with the FFD. For all 79 children, fecal, urine and buccal swab samples were collected before (t1) and after (t2) the diet intervention (Table 1). Blood sample sets (t1 + t2) for metabolite profiling and peripheral blood mononuclear cell (PBMC) isolation were complete for 76 and 67 children, respectively.

**Table 1.** Overview of collected samples and data sets.

View	Source	Samples (*n*)			Features (*n*)	
		t1	t2	t1 & t2	Total	MOFA
16S (V3–V4; genera)	Feces	79	79	79	210	210
Metagenomics species	Feces	79	79	79	332	332
Metagenomics gene families	Feces	79	79	79	2967555	4999
Plasma metabolites (named/unnamed)	Blood	77	76	76	852/215	852/215
Urine metabolites (named/unnamed)	Urine	79	79	79	904/421	904/421
Gene expression PBMCs (genes)^a^	Blood	74	71	67	19176	5003
SNPs	Buccal swabs	79	79	79	654027	4785
DNA methylation (loci)	Buccal swabs	71	77	70	850000	5000

^a^ With at least 1 mapped read in 1 sample.

We obtained eight omics datasets from fecal, urine, and buccal swabs and blood samples. Gut microbiome composition and functionality were derived from analysis of next-generation sequencing results (shotgun sequencing and 16S profiling) for DNA extracted from the fecal samples. Metabolite levels were measured in plasma and urine samples. Gene expression profiles were obtained from blood-derived PBMCs. The SNPs and methylated DNA loci profiles were derived from the buccal swabs (Table 1). The ADHD rating scale (ARS) was assessed during the screening (t0), at t1 and at t2, showing consistent scores between t0 and t1^10^, while ARS score decreased considerably from t1 to t2 (Figure 2B; Supplementary Table ST1). Of the 79 children, 50 (63%) showed a reduction in ARS score of ≥40% and were therefore categorized as responders.^16^

### Phenylalanine and tyrosine metabolism

In the published study protocol, we proposed that after adhering to FFD, there might be correlation between ARS score and levels of specific metabolites that had been previously associated with ADHD.^16^ We assessed potential associations between ARS change (categorical and continuous) and the subset of data defined as primary outcome measures in the study protocol: phenylalanine and tyrosine levels in blood and urine, and abundance of 21 gut microbial genes involved in phenylalanine and tyrosine metabolism.^16^ Spearman rank analyses (continuous) and Mann-Whitney tests (categorical) did not reveal a significant correlation of the ARS change with either the gene relative abundances measured with fecal metagenomics sequencing, or the selected metabolite levels in urine or blood (Supplementary Tables ST2 and ST3).

### Diet type affects metabolites and microbiota composition

To find cross-omics variation associated with relative change in ADHD symptoms (ARS change) between pre- and post-FFD timepoints, we employed the Multi Omics Factor Analysis (MOFA) method. MOFA can be viewed as a versatile and statistically rigorous generalization of principal component analysis (PCA) to multi‐omics data, separating the omics features into factors according to common variation.^17^ Eight data sets (referred to as “views” in the context of MOFA) were used, with a maximum of ~5000 features per view (Table 1).

**Figure 3.**
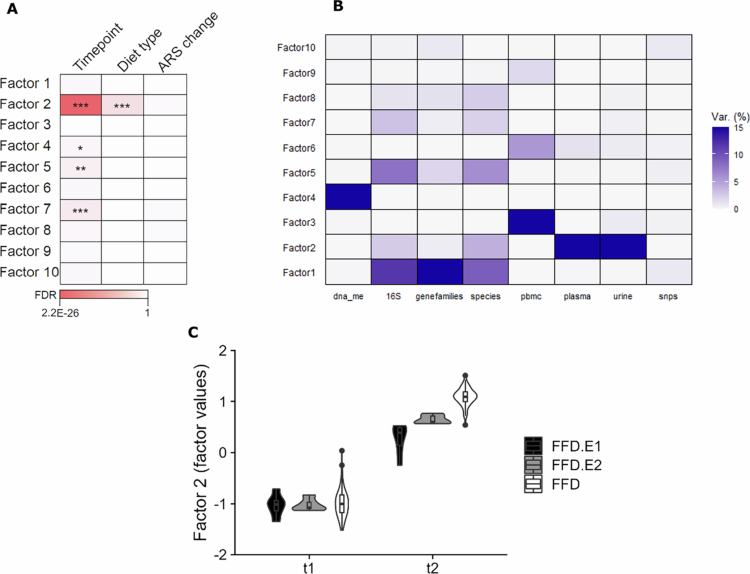
Three panel figure illustrating the 10-factor MOFA model (n = 79) results and associations. Panel A shows Associations of the 10 factors in the MOFA model with timepoint (t1 vs t2), diet type (FFD.E1, FFD.E2 or FFD) and ARS change (%). Panel B shows the explained variation (%) of the data views per factor; the views represented are DNA methylation (dna_me), 16S rRNA metataxonomic analysis (16S), shotgun metagenome gene family abundances (gene families), shotgun metagenomic species abundance (species), PBMC transcriptome gene expression values (pbmc), plasma metabolites (plasma), urine metabolites (urine), and single nucleotide polymorphisms (snps). Panel C shows Factor 2 value changes between t1 and t2 for the 3 diet types.

**Figure 4.**
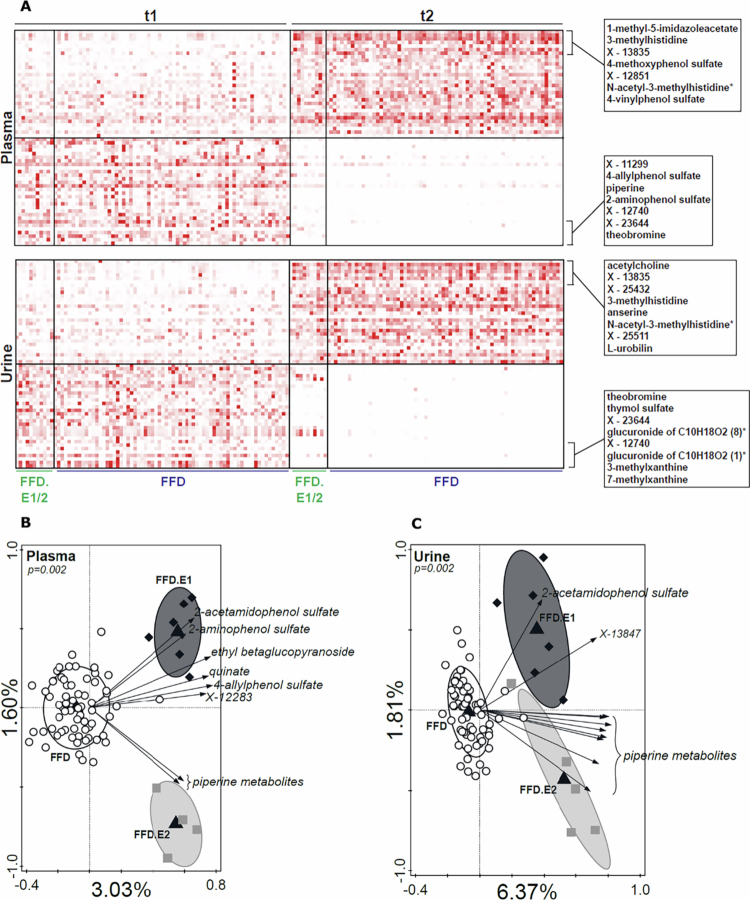
Three panel figure: heatmap and two redundancy analysis plots detailing changes in metabolite levels in plasma and urine after following the FFD. Panel A shows a heatmap of metabolite levels in blood and urine that showed the largest contribution to MOFA factor 2. Unidentified metabolites were assigned X-numbers by Metabolon. Panel B and C show RDA analyses of plasma (panel B) and urine (panel C) metabolites with diet type as an explanatory variable at t2.

We assessed whether any of the 10 factors in the MOFA model associated with ARS change, timepoint (t1 vs t2) or type of diet (FFD.E1, FFD.E2 or FFD; Figure 3A). Both the timepoint (i.e., the pre- and post-intervention measurements) and the diet type were significantly associated with factor 2, while none of the factors correlated with ARS change. Microbiome and metabolite features provided the largest contribution to the factor values in factor 2 (Figure 3B). The difference between the t1 and t2 factor 2 values indicated that the features of this factor had changed considerably between t1 and t2 (Figure 3C). The effect was largest for the FFD and smallest for the FFD.E1, suggesting that the foods that were allowed in the diet affected the metabolites and microbiome features in this factor. Several metabolites with higher levels at t2 compared to t1 were related to the consumption of poultry (1-methyl-5-imidazoleacetate, anserine, (N-acetyl-)3-methylhistidine; Figure 4A), concurrent with the FFD, during which only turkey meat is allowed (FFD.E1/E2 also allows lamb). Metabolites that were detected at t1 but not at t2 are often derived from foods not allowed during the FFD (i.e., theobromine [chocolate], thymol sulfate [herbs], 3/7-methylxanthine [caffeinated foods/drinks], and piperine [pepper]). Comparing the three diet types by RDA further showed that cereal-derived metabolites (2-aminophenol and 2-acetamidophenol) were enriched in children who followed the FFD.E1 diet type that allowed the consumption of wheat-based products (Figure 4B and C), which were eliminated from the diet when progressing to FFD.E2 and FFD diet types. Piperine metabolites were detected in both FFD.E1 and FFD.E2 children, which is in agreement with pepper being allowed in both of these diet types, while being excluded from the FFD. The homogeneous change in factor values of factor 2 coinciding with the change in diet, as well as the discriminating metabolites related to dietary intake, are further corroborating that compliance with the dietary instructions by the participants was high.

Because children that responded favorably to the FFD.E1/E2 diets all showed a high ARS change (Figure 2B) and had a smaller shift in factor 2 values from t1 to t2 compared to the FFD diet types (Figure 3C), diet type could have confounded the analyses between the MOFA factors and the ARS change. Therefore, the differentiation of different diets was consistently taken into account in downstream analysis, e.g., by exclusively analyzing the full FFD group. For example, we generated MOFA models using only the data of the children who followed the FFD (Supplementary Figure SF1). Like in the models with all children, factor 2 captured the t1‒t2 change in metabolites; however, none of the MOFA factors, including factor 2, were significantly associated with the change in ARS.

### ARS changes correlated with gut microbiome composition

**Figure 5.**
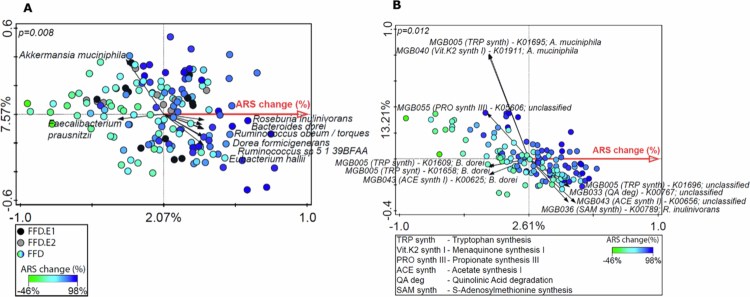
A two panel plot showing the association between the microbiome taxonomy and gut-brain module abundances and ARS change. Microbiome data from children who followed the full FFD (n = 68) determined the ordination space, to which the children on the extended (E1 and E2) diets were added as supplementary datapoints. Panel A shows the RDA using relative gut microbiome species abundance at t1 and t2 as a response variable, with ARS change as an explanatory variable and panel B shows the relative abundance of gut–brain modules (abbreviated as MGB in the figure) KOs stratified to the gut microbiome species as a response variable, with ARS change as an explanatory variable. In both plots, the axis labels depict the percentage explained variation of the RDA-axis (x-axis) and the first PCA axis (y-axis). Permutation testing was performed between subjects rather than between samples to determine the significance of the association (Panel A *p-*value = 0.008, Panel B *p-*value *= *0.012), which allows for the inclusion of two samples per child without overestimating the significance. The arrows and their corresponding labels display the most discriminant response variables driving the RDA axis separation (x-axis) in both plots.

Potential associations between ARS change and the variation in the omics data views were assessed using a supervised RDA approach. RDA analyses were performed using only the data derived from the children who followed the FFD diet type; children following the FFD.E1 and FFD.E2 were added supplementary in the RDA plot. Both the t1 and t2 timepoints were included, permuting per participant. Species abundance was significantly associated with ARS change (p = 0.008, explained variation = 2.07%, Figure 5A, Table 2). The supplementary FFD.E1 and FFD.E2 samples appeared scattered across the RDA ordination, while on the basis of the high ARS change associated with these samples, they would have been expected to be positioned towards the far-right-hand side of the plot. Therefore, the observed association between species abundance and the ARS holds for the children who responded to the FFD but not for the children who responded already to FFD-E1 or FFD-E2, justifying the separate investigation of the FFD group. Within the latter group, species such as *Bacteroides dorei*, *Akkermansia municiphila*, and *Alistipes onderdonkii* were more abundant in children with a low ARS change, while *Dorea formicigenerans*, *Roseburia inulinivorans* and *Ruminococcus torques* were more abundant in children with a high ARS. Performing RDAs per timepoint confirmed that the contributions (horizontal direction arrows) in separate RDAs for t1 and t2 were very similar (Supplementary Figure SF2).

**Table 2.** RDA results with ARS change as explanatory variable and the omics data views as response variables.

Data view	Timepoint	*N* samples	Explained variation (%)	*p*-value^a^	FDR^b^
16S (V3–V4)	t1 + t2	136	1.94	0.012	0.048
Metagenomics, species abundance	t1 + t2	136	2.07	0.008	0.048
Metagenomics, gene families abundance	t1 + t2	136	0.72	0.572	0.654
Plasma metabolites	t1 + t2	132	1.01	0.45	0.653
Urine metabolites	t1 + t2	136	0.87	0.49	0.653
SNPs	t1	68	1.42	0.358	0.653
DNA methylation	t1 + t2	126	1.42	0.104	0.277
PBMCs gene expression	t1 + t2	126	0.72	0.662	0.662

FDR = false discovery rate; SNP = single nucleotide polymorphism; PBMC = peripheral blood mononuclear cell.

^a^ Monte Carlo permutation test, Canoco5.

^b^ Benjamini & Hochberg.

To predict the functional impact of the microbial species identified, we employed gut–brain modules (GBM) that represent gut–microbial pathways with potential neuroactive effects.^18^ The abundance of the genes that make up the modules, which were stratified according to species, was used to test whether the species associated with the ARS changes represented specific modules. RDA with the stratified genes as the response variable and the ARS change as the explanatory variable found a significant association (p = 0.012, explained variation = 2.61%, Figure 5B). The observation that the explained variation was higher (difference is 0.54%) than the RDA with species abundance as a response variable suggests that functionality adds information that is relevant to the microbiome variation related to ARS change.

The contribution (RDA-axis, i.e., horizontal direction arrows) of the stratified genes to the RDA ordination shows that most genes have both positive and negative coordinates (i.e., these stratified genes were enriched in microbiota samples from children with high and low ARS changes, respectively) depending on the species by which they are encoded (Supplementary Figure SF3A). Likewise, comparing the RDA-axis contributions of the microbial species between the species RDA and stratified Kegg Orthology (KO) RDA shows that specific species can be enriched both in high and low responders, depending on the genes that they encode (Supplementary Figure SF3B). Strikingly, the taxon-specific abundances of the pathways for quinolinic acid degradation and S-adenosylmethionine synthesis were positively associated with an increased ARS response, whereas the association with the tryptophan synthesis pathway was divergently associated with the magnitude of the ARS response depending on the species (taxons) encoding the pathway. We discuss the possible links between these observations and ADHD in the discussion of this manuscript.

### Peripheral changes related to ARS change

We subsequently investigated whether the change in the ARS score (Figure 2B) was associated with changes in blood metabolite levels and PBMC transcription profiles, using EdgeR and Ingenuity Pathway Analysis (IPA). These analyses revealed only modest correlations with differential (increased) gene expression and metabolite concentrations associated with mitochondrial respiration (see Supplementary results), suggesting that the microbiome association with ARS score changes are not clearly reflected in the blood samples collected.

### Association between microbiome composition, behavior and brain activation

**Figure 6.**
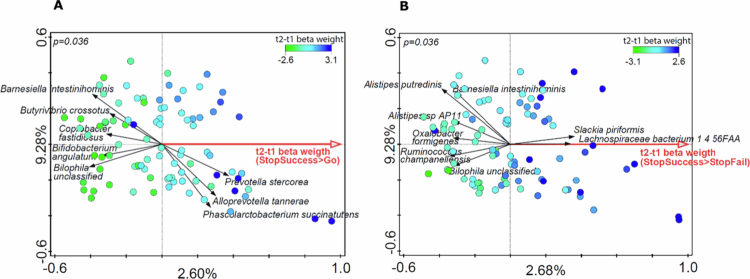
Two panel RDA plots showing the association between precuneus activation after FFD and microbiome species abundance (n = 45). Both RDAs used microbiome species abundance (measured at both timepoints) as response variables and precuneus t2–t1 beta weights of the StopSuccess > Go (panel A) or the StopSuccess > StopFail (panel B) contrasts. The axis labels depict the percentage explained variation of the RDA-axis (x-axis) and the first PCA axis (y-axis). Permutation testing was performed between subjects rather than between samples to determine the significance of the association (Panel A *p-*value = 0.036, Panel B *p-*value * = *0.036), which allows for the inclusion of two samples per child without overestimating the significance. The arrows and their corresponding labels display the most discriminant response variables driving the RDA axis separation (x-axis) in both plots.

Finally, we assessed the relationships among behavior, microbiome composition and brain activation. In our previous publication,^10^ we showed that the ARS change was positively correlated with the activation of the precuneus during the execution of a stop-signal task, applying two response inhibition contrasts (StopSuccess > Go: general linear model [GLM], df = 50, *n* = 53, pFWE = 0.015; StopSuccess > StopFail: GLM, df = 50, *n* = 53, pFWE < 0.001). To investigate whether the ARS change and correlated precuneus activation were also associated with microbiome species composition, the cluster-averaged beta weights for both response-inhibition contrasts were used as explanatory variables in RDAs, with the relative gut microbiome species composition as the response variable (Figure 6A and B). RDA found that Beta weights of precuneus activation in both contrasts were significantly associated with species relative abundance and these were similar to the species associated with ARS change (Supplementary Figure SF4).

## Discussion

FFD intervention studies^7-9^ in children with ADHD have quite consistently demonstrated substantial and clinically relevant behavior improvements in more than half of the participating individuals. However, several limitations in the study design of these studies have been raised, including the open-label approach, the lack of an appropriate placebo group, the changes in lifestyle structuring during the demanding diet, and the potential bias in parent-dependent behavior symptom rating.^19-21^ Importantly, the present study did not have the aim to evidence the behavioral benefits that can be achieved by an FFD diet, but rather assumed and confirmed that the behavioral effects in this study closely resemble those that were previously reported.^6^ The aim of this study was to unravel the MGB mechanisms potentially underlying the reduction in ADHD symptoms in children with ADHD after following an FFD. To this end, the study employs the quantitative variation in behavioral improvement (ARS-change, used both categorical and continuous) among the participants to identify correlated variations in molecular measurements obtained for the participating children before and after the intervention. As a consequence, the lack of a parallel placebo intervention, the open-label character of the study, as well as potential lifestyle structuring effects are much less relevant in this study as compared to those intending to demonstrate the efficacy of the diet in changing behavior. Nevertheless, independent and unbiased behavior ratings (rather than those reported by parents) could have strengthened the reliability and lack of bias in the ARS values obtained in this study^8^ but unfortunately were not feasible to implement in the study design.

The primary objective of this study was to hypothesize an association between the metabolite levels of tyrosine and phenylalanine and/or the relative abundances of the 21 microbial enzymes involved in their metabolism and changes in ADHD symptoms after following the diet. However, no significant associations were found, rejecting the primary hypothesis. The secondary objective was an explorative analysis of a wide range of omics data and ADHD symptom reduction (ARS response) during diet intervention. These analyses revealed that the composition of the gut microbiome was associated with the ARS response as well as our previously published activation of the precuneus brain region. Importantly, this microbiome association was identified for both the before and after intervention timepoints, indicating that it is the composition rather than a diet-induced change.

Analysis of the data revealed a substantial impact of the diet on specific metabolites and the relative abundance of specific gut microbiota taxa. Notably, the three different FFD types each presented a signature effect on blood and urine metabolites, with changes in specific metabolites that could be related to the foods allowed or excluded in the respective FFD types. The changes in the levels of these discriminating metabolites in children after following the three diet types indicate that compliance with the dietary instructions by the participants was high. To exclude a confounding effect of the diet type followed by the children, subsequent analyses included only the 68 children who followed the FFD (excluding the 11 children on the FFD.E1/2). While the unsupervised multi-omics factor analysis (MOFA) approach did not find associations between changes in ADHD symptoms and the eight data sets derived from 8 different omics types, applying the supervised RDA method on the individual data sets showed an association between ADHD symptom changes and the gut microbiome species composition. This may be interpreted to suggest that the variation in the omics data that is associated with behavior change had been too small to be detected using unsupervised methods, presumably because of genetic and molecular variation between individual children. Children who followed the FFD.E1/2 did not comply with this association, justifying separate statistical investigations by the RDA of only the children who followed the FFD. Interestingly, the ARS change associated species composition was very similar before and after the following the FFD. These results suggest that the gut microbiome plays a role in food-associated ADHD and that replacing ADHD-associated diets with an FFD had taken away dietary compounds that those bacterial proteins had acted upon or, had introduced FFD-associated compounds that improve ADHD symptoms via a microbiome‒gut‒brain axis.

Despite the apparent associations between the microbiome, brain and behavior, our gene expression data derived from peripheral blood monocyte and plasma metabolome data did not point towards peripheral factors in the blood that might mediate these microbiome‒gut‒brain axis associations. However, we cannot exclude the possibility that the timing of blood sampling disallowed the detection of such chemical messengers or associated gene expression changes. For example, food-associated metabolites that trigger ADHD symptoms may have been cleared from blood after overnight fasting and thereby remained undetected in the samples collected in this study. However, metabolite profiles obtained for urine samples collected at the same timepoint also failed to propose physiological changes associated with behavior changes, indicating that such ADHD-triggering transient food intake-associated metabolites also did not leave a footprint in urine. An alternative explanation of the behavioral changes because of the FFD diet could involve the enteric nervous system (ENS), which could react to local, mucosal changes in metabolites or gene expression that affect ENS activity in a way that influences brain activity (e.g., precuneus activation via the nervus vagus) and ADHD symptoms.

We expect that human individuality, i.e., genetic and molecular physiological differences (including microbiome) between the children has complicated identification of factors that consistently change in concert with behavioral outcome measures. For example, changes in the microbiome because of the FFD depend at least partially on the composition of the microbiome at baseline. Notwithstanding our selection criteria, standing genetic and microbiome differences between the children could have led to a loss of power to identify significant correlations between separate omics datasets and changes in ADHD symptoms after following FFDs. MOFA was designed to ameliorate this problem by looking for combinations of features (“factors”) from different omics datasets that jointly capture relatively large fractions of the total variation in the data (“principal component analysis across datasets”). While the MOFA did identify combinations of factors from multiple datasets, these combinations reflected either the impact of the diet found in blood and urine metabolomes (Figure 3B, factor 2) or correlations between microbiota composition and microbiota gene families (e.g., Figure 3B, factor 1). No factors with a significant correlation with ADHD symptom changes were found by MOFA.

The pre-diet microbiome composition is linked to the responsiveness of the participants to the FFD diet, and this signature is observed at both t1 and t2. Moreover, extensive *in silico* investigation of the metagenome revealed that the species-specific distributions of specific enzymes and pathways that were previously categorized as potential microbiome gut–brain modules (GBMs)^18^ showed significant correlations with the measured ARS changes, reflecting behavior. Intriguingly, the abundance of S-adenosylmethionine (SAM) synthesis genes (GBM036) encoded by *Roseburia inulinivorans* was positively associated with a higher ARS response, which may suggest that SAM production by these bacteria plays a role in the relationship between diet and ADHD. SAM functions as a universal methyl-donor that plays a key role in one-carbon metabolism in both prokaryotes and eukaryotes. Reduced SAM production in the gut microbiome has been proposed to affect various host-processes, including epithelial integrity, epigenetic modifications, and the modulation of neurotransmitter production.^22^ Notably, a strain of *R. inulinivorans* is included in a multi-species probiotic (psychobiotic) product that was demonstrated to have positive effects in a rodent model of depression,^23^ although this effect was not linked to SAM production by the microbial species in the product. Finally, the effect of dietary supplementation of SAM in adults with ADHD has been explored in clinical studies, generating mixed results, where one study reported on significant symptom reduction,^24^ whereas another study reported no effects.^25^ Taken together, it is tempting to speculate that SAM-production by *R. inulinivorans* positively contributes to ADHD symptom reduction. Notably, such a contribution would be diet dependent because the abundance of the SAM pathway in *R. inulinivorans* was observed at both t1 and t2, which is possibly explained through diet induced changes in the *in situ* environmental conditions in the gut at t2 liberating the SAM-producing pathway in this bacterium. The abundance of the quinolinic acid degradation pathway (GBM033; unclassified, indicating that the encoding species is unknown at this stage) in the gut microbiome is also positively associated with higher ARS responses in the participating children. The immune-regulating metabolite kynurenine, which is produced from tryptophane via the indoleamine 2,3-dioxygenase (IDO) pathway, is the precursor for quinolinic acid production via the kynurenine mono-oxygenase (KMO) and kynureninase (KYNU) pathway. Interestingly, the levels of several tryptophan- and kynurenine-derived metabolites were different in blood in ADHD individuals compared to neurotypical controls,^26^^,^^27^ and methylphenidate administration in ADHD individuals could be shown to ameliorate the homeostatic balance of these metabolites.^27^ Although the levels of quinolinic acid in blood were not changed in ADHD or upon methylphenidate treatment, urinary excretion of this metabolite was found to be elevated in ADHD individuals and was normalized upon methylphenidate treatment.^27^ These findings suggest that enhanced capacity for quinolinic acid degradation in the gut could contribute to the normalization of this neurotoxic compound in children with ADHD who do not receive methylphenidate treatment. As discussed above, the notion that the increased microbiome abundance of the (unclassified) GBM033 pathway is recognized at t1 and t2 would suggest that the diet-induced changes in the environmental conditions in the gut would have activated this pathway rather than driven its increasing abundance. Following the recognition of quinolinic acid as one of the pathways that is differentially abundant in an unclassified taxon in the microbiome, it is quite intriguing that the abundance of the tryptophan synthesis pathway (GBM005) divergently associates with the ARS response, depending on the species encoding the pathway. Whereas the metagenomic abundances of the GBM005-associated genes of *Bacteroides dorei* and *Akkermansia muciniphila* were negatively associated with the ARS response, a positive association was identified with these genes encoded by an unclassified taxon. Tryptophan has been recognized as a central precursor for multiple neuro- and immune-active molecules (e.g., serotonin, melatonin, kynurenine, various indoles), and the capacity of the gut microbiome to synthesize and convert tryptophan to a variety of derivative molecules has been recognized as a major mechanism of host‒microbe communication involved in the diet‒microbiome interaction with the brain.^28-31^ A variety of interventions have studied the effects of the modulation of the dietary level of tryptophan or its derivatives in neurological disorders, including several studies with individuals (adults and children) with ADHD. The findings of studies aiming to modulate dietary tryptophan levels by either depletion or supplementation remain inconclusive,^32^^,^^33^ which appears to be in apparent agreement with the observation that the levels of aromatic amino acids (including tryptophan) in blood did not differ between individuals with ADHD compared to neurotypical controls.^34^ Nevertheless, our results suggest that tryptophan synthesis (and conversion) by distinct microbial groups within the microbiome may play a role in the FFD effects on behavior. Particularly the combination of the association of tryptophan synthesis and quinolinic acid is intriguing because these pathways are interconnected through kynurenine and its derivatization. Importantly, bacterial tryptophan synthesis and metabolism is strictly regulated, involving a variety of mechanisms (e.g., transcriptional repression, activation, TRAP or T-box antitermination) that can respond differently to specific environmental conditions.^35^ This may suggest that GBM005 activity in the different taxa is differentially responsive to the environmental conditions in the gut that are induced by the FFD diet. Overall, this study identified several GBMs abundances (observed at t1 and t2) that could be associated with the behavior effects in a diet-dependent manner, where the diet does not seem to affect their abundance, but rather changes the in situ intestinal conditions that affect their (species-specific) expression and activity. This proposed connection between the GBMs identified remains speculative, but the underlying rationale implies that overall GBM abundance in a microbiome may not adequately predict the module's activity level. Activity levels are likely to be regulated in response to specific gut physiological and environmental conditions in a species-specific manner, which is able to change the overall metabolic output of the microbial ecosystem, as has been demonstrated in simplified models of gut-related microbial community models.^36^^,^^37^

The strength of the present study is that it confirms earlier reported beneficial effects of FFD diets on ADHD-behavioral symptoms in children, suggesting that at least part of childhood ADHD could be diet induced and, as such, potentially remedied by diet adjustment. However, future studies are essential to test causal relations. Moreover, our study illustrates that omics data sets can be used to identify molecular signatures associated with diet-induced changes in (ADHD) behavior, implying a role for the gut microbiome in FFD responsiveness. This signature could enable stratification of participants in future studies, with the intent to predict their response. Moreover, the possible involvement of specific GBMs in specific species could inspire strategies to enhance FFD-efficacy in less responsive children, e.g., providing the *R. inulinivorans* containing psychobiotic supplements to children that have a low intestinal abundance of this species.

## Methods

### Study design

This study complies with the principles of the Declaration of Helsinki (adopted by the 18th World Medical Association (WMA) General Assembly, Helsinki, Finland, June 1964, and lastly amended by the 64th WMA General Assembly, Fortaleza, Brazil, October 2013) and with the Medical Research Involving Human Subjects Act. The study was approved by the Medical Research and Ethics Committee of Wageningen University (NL63851.081.17, application 17/24, 1 February 2018) and was registered on ClinicalTrials.gov (number NCT03440346, February 2018).

The study design of the BRAIN study has been described in detail in previous publications.^10^^,^^16^ Briefly, after the intake eligible children were invited for the screening session (t0) at Wageningen University and Research, the Netherlands. Children meeting the inclusion criteria started with a two-week baseline period adhering to their regular diet, while parents documented their behavior and kept a qualitative food intake diary. After the baseline period (i.e., at t1), blood, urine, stool and buccal swab samples were collected, and ADHD symptom scores were assessed. A one-week transition period followed after t1, gradually adapting the diet to habituate to a different eating pattern. After the transition week, the children started with an extended version of the FFD (FFD.E1), allowing lamb, butter and small portions of wheat, corn, potatoes, some fruits, and honey.^7^^,^^38^ If no substantial behavioral improvement was reported, the FFD.E1 was adapted to FFD.E2 and FFD by gradually removing the additional allowed foods. The most stringent FFD consisted of rice, turkey, vegetables (cabbage [white, green, Chinese, red], beet, cauliflower, borecole, swede, sprouts, and lettuce), pears, olive oil, ghee, salt, and rice drink with added calcium and water.^39^^,^^40^ At t2, blood, urine, stool and buccal swab samples were collected, and ADHD symptom score assessments were repeated.

### Participants

This study included 79 participants (pseudo-anonymized by P-code) from the BRAIN study.^10^^,^^16^ Right-handed boys, aged ≥8 and ≤10 y, and meeting the Diagnostic and Statistical Manual of Mental Disorders, fourth edition (DSM-IV) criteria for ADHD were recruited via the media and healthcare institutions. The exclusion criteria were (i) diagnosis of autism spectrum disorder, developmental coordination disorder, chronic gastrointestinal disorder, autoimmune disorder, dyslexia, or dyscalculia; (ii) premature birth (<36 weeks) and/or known oxygen deprivation during birth; (iii) vegetarian/vegan; (iv) IQ <85; (v) use of systemic antibiotics, antifungals, antivirals or antiparasitics in the past six months; (vi) insufficient command of the Dutch language by either parents or child, (vii) family circumstances that may compromise compliance; and (viii) having a contraindication to MRI scanning. Use of other medication during the trial was recorded. The participants were allowed to withdraw from the study at any time. All parents provided written informed consent, and all the children provided written assent. The required sample size for the primary hypothesis (i.e., association between the ARS change and relative abundance of 21 gut microbial ECs and tyrosine and phenylalanine levels in blood plasma and urine) of this manuscript was estimated at 46.^16^

### Behavior scores

ADHD symptom scores were measured using the ADHD Rating Scale (ARS), which consists of 18 ADHD symptoms with a maximum score of 54.^41^^,^^42^ The ARS was completed by the parents at t0, t1 and t2, with a focus on the child’s behavior during the past week. The child’s ARS response to the FFD was determined by the percentage change in the ARS score at t2 relative to t1 (100 × [t1 − t2]/t1). To estimate the response frequency to FFD, children with an ARS change ≥40% were designated responders, whereas children with <40% ARS change were designated non-responders.^16^ Notably, in most statistical and molecular analyses reported in this manuscript, the actual response percentages per child were used rather than the responder/non-responder classification.

### Sample collection and processing

Fecal samples were collected at t0, t1 and t2. The samples were collected in DNA/RNA shield fecal collection tubes (Zymo Research/BaseClear, Leiden, The Netherlands) and kept at room temperature until storage at −80 °C. DNA was extracted according to the standard operating procedure of Knudsen et al. 2016 (https://dx.doi.org/10.6084/m9.figshare.3475406), using the QIAmp Fast DNA Stool Mini Kit (Qiagen, Venlo, The Netherlands) and Lysing Matrix B beads (MP Biomedicals, Amsterdam, The Netherlands). DNA samples were frozen and shipped to Novogene (Cambridge, United Kingdom). For all the samples, metataxonomic analyses were performed by V3–V4 16S rDNA amplicon sequencing using Illumina NovaSeq, PE150 (30000 raw tags/sample). Metataxonomic 16S sequencing reads were processed with the R package DADA2 ^43^ and taxonomically assigned using the SILVA database v132.^44^ Amplicon sequence variants (ASVs) with the taxonomic assignments “eukaryote” and “chloroplast” were discarded. For the samples obtained at t1 and t2 (i.e., directly before and after the diet intervention period), shotgun metagenome datasets were generated using Illumina NovaSeq PE250 (40 million reads/sample). Shotgun metagenome sequence reads were processed with HUManN2^45^ (v2.8.1). Relative bacterial species abundance, as determined by Metaphlan 2.0, and uniref90 gene families counts per million (CPM) were used for analyses. Unmapped reads (~37% of total reads/sample) were removed before renormalization to CPM. In addition, for each sample, reads were assembled into contigs with SPAdes version 3.14.1^46^ using the – meta option. Coverage was determined by mapping the reads back to these contigs as custom database in HUManN2. Abundance of the KOs that make up the proposed gut–brain modules (GBMs)^18^ were determined through HMM (hidden Markov model) search using HMMER 3.1b2 (http://hmmer.org/) against KOfam (HMM profiles for KEGG/KO with predefined score thresholds, https://www.genome.jp/ftp/db/kofam, downloaded on 26Aug2021). The abundances of GBM KOs stratified to gut microbial species were determined by regrouping the HUManN2 gene abundance table to KO abundance, keeping the taxa stratification.

Blood plasma and urine samples were collected at t1 and t2 and were available for 77 (t1) and 76 (t2) children. For 76 children, both t1 and t2 samples were available. Blood samples were collected in BD Vacutainer® EDTA tubes (BD Biosciences, Vianen, The Netherlands), and plasma was isolated by centrifugation at 1300 × *g* for 10 min. Urine was collected and stored at −80 °C. The plasma and urine samples were shipped to Metabolon, Inc. (Morrisville, United States) for small-molecule profiling (HD4 Global Metabolomics platform).

Blood samples for PBMC isolation were collected at t1 and t2 and were available for 77 (t1) and 73 (t2) children. For 76 children, both t1 and t2 samples were available. Blood samples for PBMC isolation were collected using BD Vacutainer® CPT™ Mononuclear Cell Preparation Tube – Sodium Citrate (BD Biosciences, Vianen, The Netherlands). PBMCs were isolated according to the manufacturer's protocol, resuspended in TRIzol (Invitrogen) and stored at −80 °C. RNA was extracted according to the manufacturer's protocol, resuspended in H_2_O and stored at −80 °C. The RNA samples were shipped to Novogene (Cambridge, United Kingdom) for ribo-depleted RNA sequencing (Illumina NovaSeq, PE150, 40 million raw reads/sample). The sequencing data was processed with the nf-core/rnaseq pipeline (10.5281/zenodo.1400710),^47^ applying the STAR-salmon option and mapping to the reference genome GRCh37.

Buccal swaps were collected at t1 and t2 using the Puritan™ HydraFlock™ Flocked Swabs (Merck Life Science NV, Amsterdam, The Netherlands). The collected samples were stored at −80 °C. The samples were transported to the HuGe-F facility of the Erasmus MC (Rotterdam, The Netherlands), where DNA methylation and SNP profiles were generated using the Infinium MethylationEPIC v1.0 (Legacy BeadChip, B5) and the Infinium™ Global Screening Array-24 v3.0 BeadChip, respectively. The Infinium MethylationEPIC v1.0 data was processed using the R package meffil.^48^

L-codes are individual samples linked to P-codes that were assigned to individual participants (see supplementary datafile SDF1).

### Data selection and preprocessing

For the primary objective, the abundances of the 21 gut microbial enzymes involved in tyrosine and phenylalanine metabolism selected on EC number (1.10.3.1, 1.14.16.1, 1.14.18.1, 1.3.1.43, 1.3.1.78, 1.3.1.79, 1.4.1.20, 1.4.3.2, 2.6.1.1, 2.6.1.5, 2.6.1.57, 2.6.1.58, 2.6.1.9, 4.1.1.25, 4.1.1.28, 4.1.99.2, 4.2.1.51, 4.2.1.91, 4.3.1.23, 4.3.1.24, 5.4.3.6) were used. In addition, the metabolites levels in blood and urine of phenylalanine and tyrosine and their derivatives were collected from the metabolon data. Data was used without further preprocessing.

For the secondary, explorative objective, all available data was included in the per-dataset multivariate analysis. For the MOFA analysis, datasets were reduced to have ~5000 features or fewer, which required the filtering of four data sets. (1) The fecal metagenomics gene families table was reduced from 2967553 to 4999 based on prevalence and CPM (gene families with <42 CPM in <20 samples were removed). (2) The PBMC RNA expression data was reduced from 57774 to 5003 genes based on prevalence and TPM (transcripts per million) (length scaled) [transcripts with <18 TPM in <13 samples were removed]. (3) The SNP data table was reduced from 654027 to 4785 features based on classification and prevalence [SNPs were removed from the table when they did not meet the two filtering criteria: (i) being classified as nonsense or missense and (ii) prevalence of the non-dominate allele in ≥10 participants]. (4) The DNA methylation data table was reduced from 863560 to 5000 features based on the coefficient of variation (cv) for each feature, selecting the 5000 features with the highest cv (leading to the removal of all methylation features with a cv <0.62).

### Statistical analyses

For the primary study outcome, *p*-values were corrected for multiple testing based on the number of genes and metabolites tested. For the explorative secondary analyses, individual bivariate analyses were FDR corrected within the dataset, while for explorative multivariate analyses (where each analysis provides a single *p*-value), these were jointly corrected for multiple testing. In all analysis, ADHD subtypes (hyperactive, inattentive; supplementary datafile SDF1) were analyzed independently, but this never yielded a stronger or more significant result than the overall ARS. The recorded use of medication was tested as a potential confounder and explanatory variable, generating no significant results.

Spearman rank correlation analysis was performed with the R package stats. Multi-omics factor analysis was performed using the MOFA2 package.^17^^,^^49^ MOFA is an unsupervised method for integrating multi-omics data producing latent factors across datasets. The standard data, model and training options were used for all the models with the exceptions of not scaling the views and the convergence mode set to “slow”, and the data were transformed with log(100*x + 1). The number of learned factors was set to 10, according to the general MOFA guidelines (https://biofam.github.io/MOFA2/faq.html). Redundancy analysis was performed in Canoco version 5.12,^50^ with default settings. In RDAs that included both t1 and t2 timepoints, permutation was performed per participant to maintain coupling of the two measurements per individual. Differential gene expression analysis was conducted using the R package EdgeR,^51^ applying the default settings for the qCML approach to compare responders and non-responders. Differential gene expression data were analyzed using Ingenuity Pathway Analysis (IPA; QIAGEN, https://www.qiagenbioinformatics.com/products/ingenuity-pathway-analysis).^52^ IPA includes expertly curated biological interactions and functional annotations created from millions of individually modeled relationships between diverse molecular, cellular and clinical entities. The complete IPA output can be requested from the authors. As the IPA input, HMDB metabolite abundance data and Ensembl ID gene expression data after EdgeR preprocessing to obtain fold-changes and *p*-values were provided and a core analysis with default settings was performed for the urine (metabolomics) and blood plasma (metabolomics and gene expression) datasets. The output of IPA includes the overlap between the input data and (canonical) pathways, upstream regulators, diseases and cellular processes in Ingenuity's knowledge base and returns two measures of association: (1) a ratio of the number of input metabolites that map to the pathway divided by the total number of metabolites that map to the same pathway and (2) a *p*-value of the corresponding Fisher's exact test to evaluate statistical support that the pathway might have been (significantly) modulated. From these and the knowledge contained in the IPA Knowledge Base, IPA calculates z-scores that provide statistical support for determining whether the modulated pathway was induced or repressed at the time of sample collection and predicts molecules (cellular genes, proteins, regulatory RNAs, metabolites, and pharmacological compounds) that might underpin the reported pathways and their corresponding z-scores. If the observed direction of change in a sufficiently large set of pathways or downstream genes (upstream regulators) is significantly consistent with a particular activation state of a pathway or transcriptional regulator, IPA predicts that the pathway or regulator has been “activated” or “inhibited”; such pathways and regulators have z-scores > 2 or <−2. To facilitate comparing the most relevant IPA outputs, we used z-scores as cut-off and listed the numbers of significantly activated or inhibited pathways and upstream regulators, with downstream target overlap at *p*-values <0.05.

## Supplementary Material

Supplementary material SDF1_BRAIN_behaviour_scores_Pcode_Lcode.xlsx

Supplementary material SDF3_BRAIN_RNAseq_tpm_scores_Lcode.xlsx

Supplementary material Supplementary Material.docx

Supplementary material SDF2_BRAIN_metabolomics_Lcode.xlsx

## Data Availability

The individual behavior scores per participant are provided in Supplementary Table ST1, as well as through supplementary datafile SDF1, and the Data Archiving and Networked Services (DANS) of the Netherlands Science Foundation (NWO); doi: 10.17026/dans-z56-fz75. The blood and urine metabolite data are available in supplementary datafile SDF2, and at DANS; doi: 10.17026/dans-xuc-jsux. The PBMC RNAseq transcriptome data are available in supplementary datafile SDF3. The sequence files underlying the 16S rRNA microbiota composition profiling are available through the European Nucleotide Archive (ENA) repository; project code PRJEB96501. The functional MRI data sets used in this study were previously published^10^ and are available at DANS; doi: 10.17026/dans-xzf-wh36. Since the remaining datasets include potential participant-traceability information, they are considered privacy-sensitive, therefore access to Shotgun metagenome, SNP profiling, and DNA-methylation data can be requested via the communicating author.
