# Supplementary material for: Microbiome signatures correlate with diet-mediated ADHD symptom reduction
Source: Gut Microbes. 2026 Apr 16;18(1):2659400. doi: 10.1080/19490976.2026.2659400 (PMC13089928; doi:10.1080/19490976.2026.2659400)
Supplement: Supplementary material — docx [file KGMI_A_2659400_SM1167.docx]

**Supplementary Material**

**Microbiome signatures correlate with diet-mediated ADHD symptom reduction**

Hontelez S.^1^, Guthrie M.^1^, Stobernack T.^1^, van Baarlen P.^1^, Rousseau C.^2^, Boks M.P.^2^, Rodrigues Pereira R.^3^, Boekhorst J.^1^, Kleerebezem M.^1*^

^1^ Host-Microbe Interactomics, Wageningen University and Research, De Elst 1, 6708 WD, Wageningen, The Netherlands. ADHD@wur.nl.

^2^ Department of Psychiatry, Brain Center University Medical Center Utrecht, University Utrecht, Utrecht, The Netherlands.

^3^ Medical Centre Kinderplein, Rotterdam, The Netherlands.

# Supplementary Results

## Peripheral changes related to ARS change

To investigate whether the change in ARS score between t1 and t2 (figure 2B) was related to changes in peripheral metabolites and gene expression, plasma metabolite profiles and gene expression levels of PBMCs at t2 corrected for baseline (t1) were investigated using the EdgeR software tool. Basically, the hypothesis that a beneficial decrease in ARS correlated significantly with differential expression of genes and/or abundance of specific metabolites was tested. When using multiple testing correction, EdgeR reported one differential metabolite (X – 25217) and no differentially expressed genes (DEGs) at pFDR<0.05. Since no significant relations were detected for individual genes and only one for the plasma metabolites, we extended the analysis to pathway enrichment, using the Ingenuity Pathway Analysis (IPA) software. IPA uses a knowledge base of more than 6 million validated interactions between genes, proteins and metabolites to calculate enrichment of pathways based on differential metabolites and gene expression data. When using as input, 770 differentially expressed genes and 25 metabolites (p[uncorrected]<0.05), IPA reported that 46 canonical pathways, involved in diverse cellular metabolic-, energy and immune-related pathways were significantly modulated (p<0.05), of which 2 were predicted (z-scores <-2 or >2; see Methods) to be repressed, i.e., representing pathways containing genes that correlated significantly with larger decreases in ARS. No pathways with z-scores >2 were reported. The 2 pathways with z-scores < 2 were “Assembly of RNA Polymerase II Complex” (p=0.021, z-score=-2.0) and “Oxidative Phosphorylation” (p=0.045, z=-2.4). IPA predicted that the observed differential gene expression patterns and the associated canonical pathways might be at least partially explained by activity of 8 activated regulators; repression of cellular pathways might have been controlled by inhibition of 10 regulators. Of these, regulators KDM5A, CEBPB and REL might have been involved in repression of pathway "Assembly or RNA polymerase II complex". These IPA results suggest that larger decreases in ARS score modestly associated with differential (increased) expression of genes and concentrations of metabolites involved in gene expression and mitochondrial respiration.

# Supplementary Tables

**Table ST1.** ADHD symptoms were assessed at three time points during the study (screening [t0], before the FFD [t1] and after 5 weeks of FFD [t2]), using the 18-item ADHD rating scale (ARS) [DuPaul, G. J. 1991. *Journal of Clinical Child Psychology* **20**, 245-253] based on the DSM-IV criteria. The ARS was completed by the parents, and ADHD symptoms were rated using a point-scoring system (0-54 points) [Pelsser, L. M. *et al.* 2011. *Lancet* **377**, 494-503]. The difference between the t1 and t2 scores determined the response to the FFD diet intervention [ARS(total)], expressed as a percentage, and categorized in response percentage classes (FFD responders: >40% reduction; FFD non-responders: <40% reduction). In addition, the partial ARS rating scores associated with inattention [ARS(inatt)] and hyperactivity [ARS(hyper)] were calculated and expressed in the same manner (see below). Data entry was performed and checked independently by two persons.
P-codes (column 1) refer to individual participants in this study. The L-codes (right-hand columns) refer to the t1 and t2 sample codes that correspond with the t1- and t2- molecular data of these individuals. To facilitate reuse of the data, this Table ST1 is also provided as **Supplementary datafile SDF1**.

*Table ST1*

**Table ST2.** Association between ARS change (responders vs non-responders) and microbial genes of the phenylalanine and tyrosine synthesis pathways and phenylalanine and tyrosine levels in urine and plasma.

|  | **Mean (sd)** | | | | **Association ARS change (parametric)^1^** | | | | **Association ARS (non-parametric)^2^** | |
| --- | --- | --- | --- | --- | --- | --- | --- | --- | --- | --- |
|  | **Responders** | | **Non-responders** | |  |  |  |  |  |  |
| **Microbial genes (EC)** | t1 | t2 | t1 | t2 | **p-value (t1)** | **p-value (ARS change[%])** | **FDR^3^ (ARS change[%])** | **p-value (shapiro-Wilk.)** | **p-value** | **FDR^3^** |
| 1.3.1.43 | 2.31 (6.16) | 4.47 (6.25) | 1.97 (3.66) | 7.04 (11.14) | 5.03E-11 | 0.54 | 0.78 | 1.57E-05 | 0.73 | 0.95 |
| 2.6.1.1 | 283.21 (33.41) | 261.63 (35.25) | 280.67 (43.47) | 253.69 (35.89) | 0.04 | 0.38 | 0.62 | 0.01 | 0.94 | 0.98 |
| 2.6.1.57 | 0.7 (1.19) | 1.5 (2.71) | 1.63 (4.01) | 1.5 (2.41) | 1.78E-08 | 0.93 | 0.93 | 0.01 | 0.93 | 0.98 |
| 2.6.1.9 | 248.75 (33.37) | 238.87 (32.42) | 250.98 (42.8) | 221.83 (29.58) | 0.07 | 0.01 | 0.13 | 5.64E-03 | 0.11 | 0.48 |
| 4.1.1.25 | 0.05 (0.25) | 0.13 (0.42) | 0.12 (0.46) | 0.11 (0.48) | 4.65E-07 | 0.15 | 0.33 | 3.68E-03 | 0.03 | 0.20 |
| 4.1.1.28 | 0.12 (0.32) | 0.3 (0.55) | 0.01 (0.04) | 0.26 (0.57) | 1.98E-03 | 0.82 | 0.92 | 1.66E-13 | 0.41 | 0.78 |
| 4.1.99.2 | 0.28 (0.48) | 0.44 (0.66) | 0.54 (1.17) | 0.2 (0.41) | 0.34 | 0.07 | 0.30 | 1.15E-10 | 0.02 | 0.20 |
| 4.2.1.51 | 97.66 (31.42) | 114.49 (33.67) | 106.36 (39.62) | 119.56 (37.86) | 6.5E-04 | 0.84 | 0.92 | 0.02 | 0.59 | 0.95 |
| 4.2.1.91 | 2.86 (2.22) | 3.63 (3.39) | 3.19 (2.38) | 3.78 (2.23) | 1.69E-12 | 0.85 | 0.92 | 0.82 | 0.98 | 0.98 |
| **Metabolites** |  |  |  |  |  |  |  |  |  |  |
| Phenylalanine |  |  |  |  |  |  |  |  |  |  |
| *Plasma* | 1.01 (0.12) | 1.00 (0.12) | 0.96 (0.09) | 1.05 (0.12) | 0.54 | 0.14 | 0.33 | 0.02 | 0.25 | 0.78 |
| *Urine* | 1.03 (0.28) | 1.13 (0.3) | 1.00 (0.24) | 1.02 (0.37) | 0.8 | 0.1 | 0.33 | 0.73 | 0.40 | 0.78 |
| Tyrosine |  |  |  |  |  |  |  |  |  |  |
| *Plasma* | 1.08 (0.18) | 0.94 (0.16) | 1.03 (0.2) | 1.01 (0.22) | 0.63 | 0.18 | 0.33 | 0.03 | 0.73 | 0.95 |
| *Urine* | 1.07 (0.36) | 1.08 (0.36) | 1.06 (0.36) | 0.93 (0.37) | 0.39 | 0.04 | 0.26 | 0.32 | 0.42 | 0.78 |

^1^ ANCOVA: t2 ~ t1 + ARS change (responder vs non-responder)

^2^ Mann-Whitney test: responders log2(t2/t1) vs non-responders log2(t2/t1)

^3^ Benjamini & Hochberg

**Table ST3.** Association between ARS change (%) and microbial genes of the phenylalanine and tyrosine synthesis pathways and phenylalanine and tyrosine levels in urine and plasma.

|  |  |  | **Association ARS change (parametric)^1^** | | | | **Association ARS (non-parametric)^2^** | |
| --- | --- | --- | --- | --- | --- | --- | --- | --- |
|  | **t1**  **Mean (sd)** | **t2**  **Mean (sd)** | **p-value (t1)** | **p-value (ARS change[%])** | **FDR^3^ (ARS change[%])** | **p-value (shapiro-Wilk.)** | **p-value** | **FDR^3^** |
| **Microbial genes (EC)** |  |  |  |  |  |  |  |  |
| 1.3.1.43 | 2.19 (5.35) | 5.41 (8.41) | 0.01 | 0.13 | 0.28 | 3.67E-10 | 0.62 | 0.90 |
| 2.6.1.1 | 282.28 (37.17) | 258.71 (35.46) | 0.16 | 0.12 | 0.28 | 0.23 | 0.96 | 0.96 |
| 2.6.1.57 | 1.04 (2.62) | 1.5 (2.59) | 0.05 | 0.64 | 0.80 | 3.20E-11 | 0.91 | 0.96 |
| 2.6.1.9 | 249.57 (36.85) | 232.61 (32.29) | 0.12 | 0.01 | 0.13 | 9.32E-06 | 0.18 | 0.59 |
| 4.1.1.25 | 0.08 (0.34) | 0.13 (0.44) | 2.98E-06 | 0.74 | 0.80 | 3.84E-16 | 0.13 | 0.56 |
| 4.1.1.28 | 0.08 (0.26) | 0.28 (0.56) | 0.01 | 0.94 | 0.94 | 2.03E-13 | 0.62 | 0.90 |
| 4.1.99.2 | 0.37 (0.81) | 0.36 (0.59) | 0.76 | 0.06 | 0.26 | 5.68E-09 | 0.02 | 0.26 |
| 4.2.1.51 | 100.85 (34.66) | 116.35 (35.11) | 1.75E-03 | 0.69 | 0.80 | 0.34 | 0.32 | 0.83 |
| 4.2.1.91 | 2.98 (2.27) | 3.68 (3.01) | 7.80E-07 | 0.50 | 0.72 | 3.17E-08 | 0.47 | 0.87 |
| **Metabolites** |  |  |  |  |  |  |  |  |
| Phenylalanine |  |  |  |  |  |  |  |  |
| *Plasma* | 0.99 (0.11) | 1.02 (0.12) | 0.53 | 0.21 | 0.36 | 0.01 | 0.08 | 0.52 |
| *Urine* | 1.02 (0.26) | 1.09 (0.33) | 0.76 | 0.22 | 0.36 | 0.47 | 0.39 | 0.85 |
| Tyrosine |  |  |  |  |  |  |  |  |
| *Plasma* | 1.06 (0.19) | 0.96 (0.19) | 0.65 | 0.08 | 0.26 | 0.03 | 0.83 | 0.96 |
| *Urine* | 1.07 (0.36) | 1.02 (0.37) | 0.44 | 0.049 | 0.26 | 0.41 | 0.80 | 0.96 |
| ^1^ ANCOVA: t2 ~ t1 + ARS change (%)  ^2^ Spearman rank: microbial genes (EC) or metabolites log2(t2/t1) vs ARS change (%)  ^3^ Benjamini & Hochberg | | | | | | | | |

# Supplementary Figures

| **A** | **B** |
| --- | --- |
| 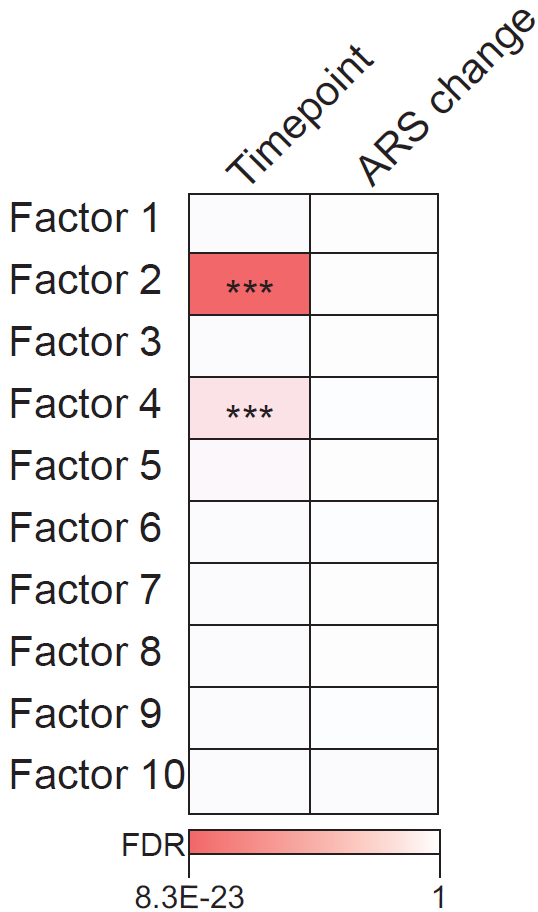 | 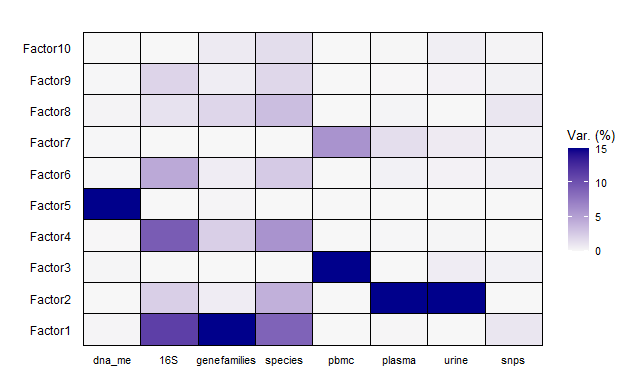 |
| **Figure SF1.** 10-factor MOFA model, FFD only (n=68). A) Associations of the 10 factors in the MOFA model with timepoint (t1 vs t2) and ARS change (%). B) Explained variation (%) of the data views per factor. | |

| 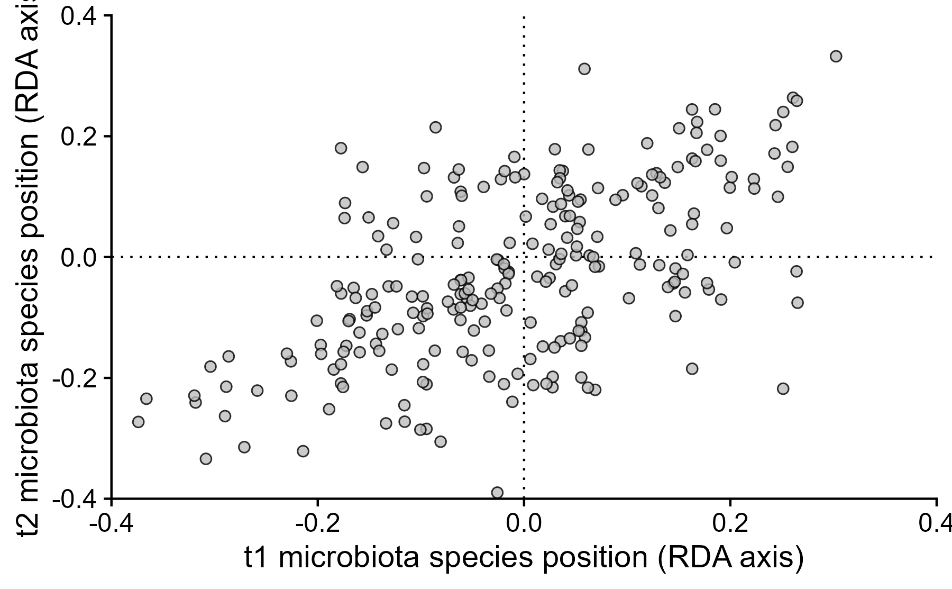 |
| --- |
| **Figure SF2.** Scatterplot of the t1 and t2 microbiome species positions on the ordination axes of the RDAs with ARS change as explanatory variable and gut microbiome species composition at t1 (p=0.042, explained variation=2.13%) or t2 (p=0.006, explained variation=2.40%) as response variables (FFD only, n=68). |

| **A** | 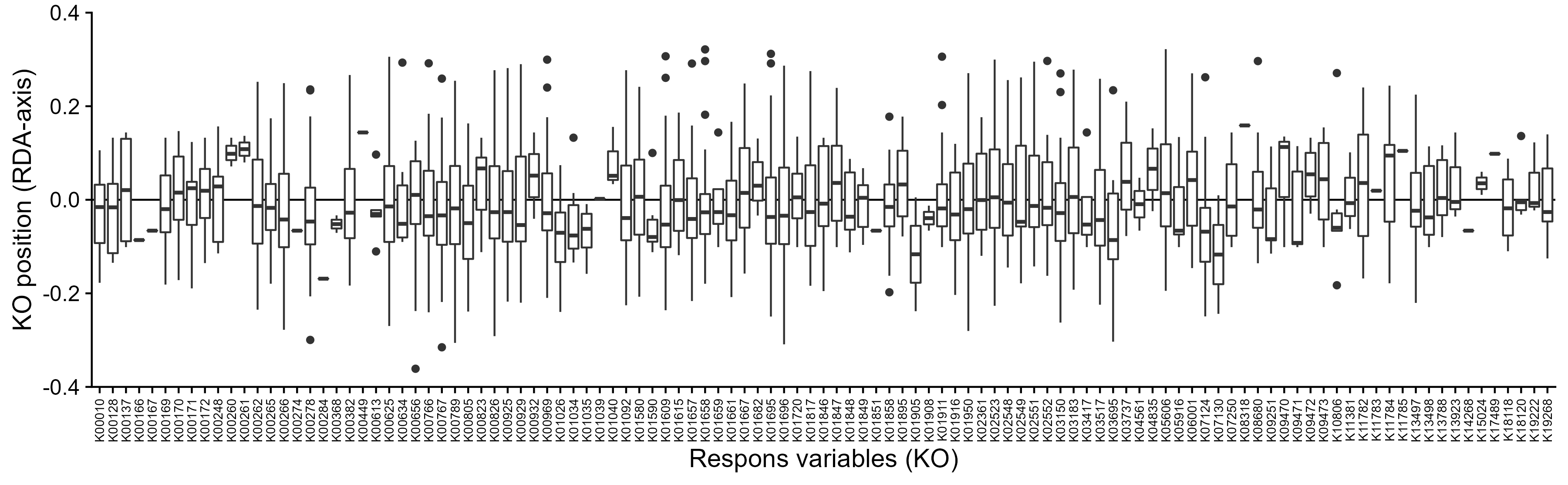 |
| --- | --- |
| **B** | 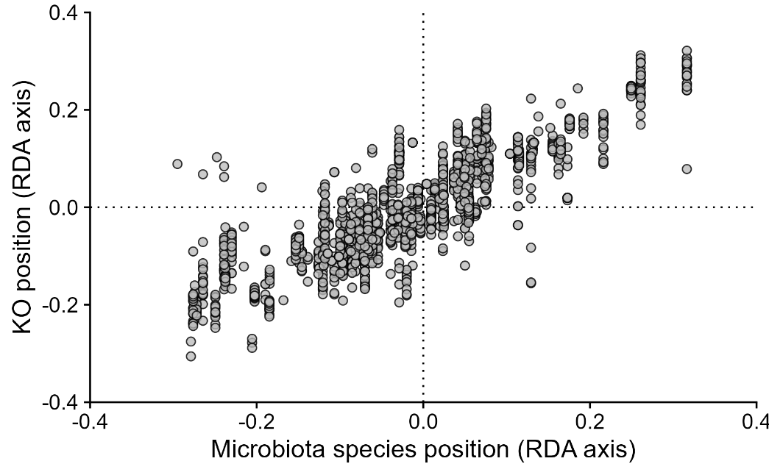 |
| **Figure SF3.** Positions of the KO response variables in the RDA ordination axis of the RDA with ARS change (%) as explanatory variable and relative KO abundance stratified to species (Figure 5B in the main manuscript) or relative species abundance (Figure 5A in the main manuscript) as response variables. A) Boxplot of KO positions per KO (x-axis) stratified to microbiota species by which they are encoded (y-axis). B) Scatterplot of KO positions stratified to microbiota species (y-axis) and species microbiota positions of the RDA with relative species abundance as response variable (x-axis). | |

| **A** | **B** |
| --- | --- |
| 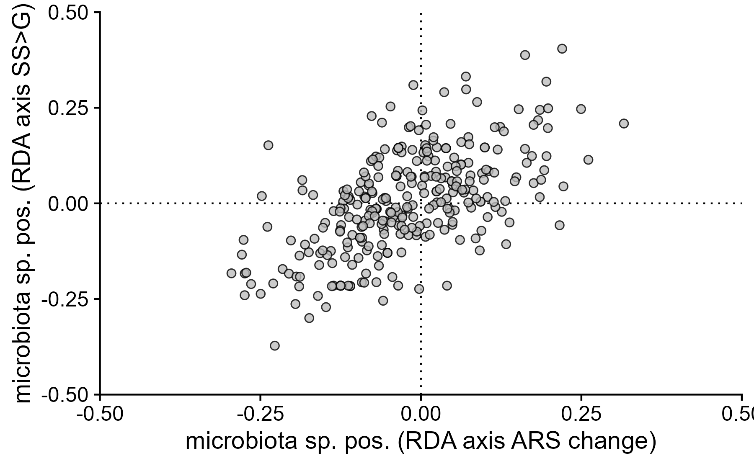 | 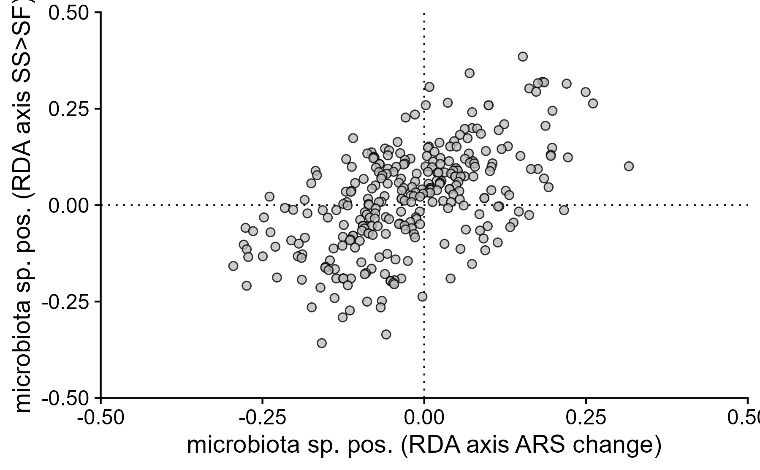 |
| **Figure SF4***.* Scatterplots of the microbiota species positions on the RDA axis in the RDAs with ARS change as explanatory variable versus t2-t1 beta weights of the A) StopSuccess>Go or B) StopSuccess>StopFail contrasts. | |
